# Exploration of the Role of Cyclophilins in Established Hepatitis B and C Infections

**DOI:** 10.3390/v17010011

**Published:** 2024-12-25

**Authors:** Jennifer Molle, Sarah Duponchel, Jennifer Rieusset, Michel Ovize, Alexander V. Ivanov, Fabien Zoulim, Birke Bartosch

**Affiliations:** 1 INSERM U1052, CNRS UMR5286, Université Claude Bernard Lyon 1, Hospices Civils de Lyon, Lyon Hepatology Institute (IHU Everest), 69003 Lyon, France; jennifer.molle@inserm.fr (J.M.); sarah.duponchel@gmail.com (S.D.); fabien.zoulim@inserm.fr (F.Z.); 2 CarMeN Laboratory, INSERM U1060, INRA U1397, Lyon Hepatology Institute, 69007 Lyon, France; jennifer.rieusset@univ-lyon1.fr (J.R.); movize@amolyt.com (M.O.); 3 Center for Precision Genome Editing and Genetic Technologies for Biomedicine, Engelhardt Institute of Molecular Biology, Russian Academy of Sciences, 119991 Moscow, Russia; aivanov@yandex.ru

**Keywords:** hepatitis virus, cyclophilin, liver, anti-viral treatment

## Abstract

Cyclophilin (Cyp) inhibitors are of clinical interest in respect to their antiviral activities in the context of many viral infections including chronic hepatitis B and C. Cyps are a group of enzymes with peptidyl-prolyl isomerase activity (PPIase), known to be required for replication of diverse viruses including hepatitis B and C viruses (HBV and HCV). Amongst the Cyp family, the molecular mechanisms underlying the antiviral effects of CypA have been investigated in detail, but potential roles of other Cyps are less well studied in the context of viral hepatitis. Furthermore, most studies investigating the role of Cyps in viral hepatitis did not investigate the potential therapeutic effects of their inhibition in already-established infections but have rather been performed in the context of neo-infections. Here, we investigated the effects of genetically silencing Cyps on persistent HCV and HBV infections. We confirm antiviral effects of CypA and CypD knock down and demonstrate novel roles for CypG and CypH in HCV replication. We show, furthermore, that CypA silencing has a modest but reproducible impact on persistent HBV infections in cultured human hepatocytes.

## 1. Introduction

Cyclophilins are a group of enzymes with peptidyl-prolyl isomerase activity (PPIase), involved in a variety of functions related to cell metabolism and energy homeostasis. Their expression is frequently enhanced in inflammation or malignancy. Cyps are thought to aid the folding and assembly of other proteins by catalyzing the isomerization of prolines, interconverting this amino acid between cis and trans isomers [1]. Amongst the 17 family members in humans, Cyclophilin A (CypA), the most abundantly expressed family member, is present mainly in the cytoplasm and is a host factor involved in the life cycle of multiple viruses. CypB and CypC have amino-terminal signal sequences that target them to the ER protein secretory pathway. CypA, B, and C can be secreted and are potent pro-inflammatory mediators [2,3,4]. CypD has a signal sequence that directs it to the mitochondria, where it is a key regulator of mitochondrial permeability transition pores and is critical for necrotic cell death. CypG and H localize to the nucleus, where they are implied in splicing and transcription [5]. 

Cyclophilins have been shown to be required for replication of diverse viruses including HIV 1 [6,7], HCV [8,9,10], Vaccinia virus [11], West Nile, Dengue, Yellow Fever [12], HBV [13], HPV [14], HCMV [15], coronaviruses [16,17], JEV [18], Influenza A [19], and VSV [20]. Cyclophilin inhibitors (CypIs) have exhibited a broad spectrum of antiviral activity. The first identified CypI was cyclosporine A (CsA), an immunosuppressant due to its capacity to decrease the activity and proliferation of T-lymphocytes by the inhibition of calcineurin in a ternary complex formed with CypA. Since then, two structurally distinct main classes of non-immunosuppressive cyclophilin inhibitors have been developed: cyclosporine A (CsA) analogs such as alisporivir, CRV431, SCY-635, NIM811, and STG-175 and the sangliferin analogs such as NV556. More recently, a complex fragment-based drug discovery approach was used to design a new family of small-molecule, nonpeptidic CypIs (SMCypIs) unrelated to CsA [21]. CypIs neutralize the PPIase activity of members of the cyclophilin family by binding to their enzymatic hydrophobic pockets. As the catalytic center of cyclophilins are highly conserved, targeting of specific Cyps with pharmacological inhibitors has been a difficult task [22]. A striking effect of CypIs was demonstrated for HCV in vitro and in vivo [23,24,25,26], but recent studies suggest that CypIs may also be effective against HBV in vitro [13,27,28,29,30] and in transgenic mice [31]. 

The molecular mechanisms underlying the antiviral effects of CypIs remain unclear. The Cyp whose role in viral replication is best studied is CypA. In respect to hepatitis viruses, CypA is known to interact with and affect the folding of hepatitis C virus (HCV) viral protein NS5A [8,10,32,33,34,35], important for double membrane vesicle formation and replication [36,37,38,39]. Interactions of CypA with the HCV polymerase NS5B increase replication [8]. CypA furthermore modulates polyprotein cleavage at the NS5A/NS5B [10] and the NS2/NS3 junctions [40]. Biochemical studies identified a direct interaction between NS5A and the isomerase active sites of CypA and CypB [33] in an RNA binding region that coincides with NS5B binding, suggesting formation of a ternary complex between CypA, NS5A, and NS5B that regulates viral RNA replication. Besides CypA, CypB, CypD, CypH, and Cyp40 have also been shown to be required for HCV replication [8,9,10,41,42,43,44,45,46]. In addition, knock out of CypD, E, H, and 40 by siRNA lowered replication efficacies of replicons [41,45,47], and for CypD, this has been confirmed with HCVcc [46,47]. CypIs such as cyclosporine A (CsA), were shown to inhibit HCV 1b and 2a replicons [42,48], while more recently, non-immunosuppressive CypIs have been described that suppress HCV replication in vitro and in vivo [49,50,51]. In the context of hepatitis B infection, CypIs have been shown to inhibit HBV infection in vitro by blocking viral entry [13,27,28,29,30]. The CsA analog alisporivir and the sanglifehrin analog NV556, as well as knock out of CypA, C, and D also reduced intracellular and secreted HBV in cell lines stably expressing or transfected with HBV [29,30], suggesting that cyclophilin members facilitate HBV replication. Together, these data indicate that Cyps are required at two distinct steps of HBV infection—entry and post-entry. Finally, the CypI CVRV431 reduced liver HBV DNA and serum HBsAg, but did not influence HBV DNA and HBeAg levels in blood, nor HBsAg levels in the liver on HBV transgenic mice [31].

Overall, the use of CypIs has shown interesting antiviral effects in vitro and in vivo, but it remains unknown what particular Cyps are involved in mediating these antiviral effects. Importantly, the role of particular Cyps in the viral life cycles of hepatitis viruses by genetic knock down has so far been predominantly tested in neo-infections but rarely been tested in the context of an already-established infection (with the exception of HCV replicon cell lines). Here, we applied genetic interference targeting Cyp A, B, C, D, G, and H to extend our knowledge of particular Cyps in the replication of hepatitis viruses; furthermore, genetic interference was applied in the context of already-established viral infections, in order to investigate which Cyps are implied in mediating antiviral effects.

## 2. Materials and Methods

### 2.1. Cell Culture

Huh7.5 and HepG2-hNTCP cells were maintained in Dulbecco’s Modified Eagle Medium containing 1X GlutaMAX (ThermoFisher Scientific, Waltham, MA, USA), 100 U/mL penicillin, and 100 µg/mL streptomycin (ThermoFisher Scientific, Waltham, MA, USA). For Huh7.5, the medium was complemented with 10% FCS (High clone, GE healthcare, Chicago, IL, USA); for HepG2-hNTCP, the medium was complemented with 5% Fetal Clone II (GE Healthcare), 1mM sodium pyruvate, and 5 µg/mL puromycin. Primary human hepatocytes (PHHs) were isolated from surgical liver resections from HBV-, HCV-, and HIV-negative adult patients undergoing lobectomy or segmental liver resection for medically required purposes unrelated to this research with informed consent (institutional review board agreements DC-2008-99 and DC-2008-101). PHHs were isolated as previously described [52] and plated in complete William’s medium supplemented with 100 U/mL penicillin, 100 µg/mL streptomycin, 2 mM glutamine, 5 mg/mL human insulin (Sigma-Aldrich/Merck St. Louis, MO, USA), 25 mg/mL hydrocortisone hemisuccinate, and 5% Fetalclone II. After 24 h, PHHs were extensively washed and kept in serum-free medium for one more day to counter select the growth of contaminating fibroblast and endothelial cells and were infected 48 h after plating with HBV virus (i.e., inoculum).

### 2.2. Viral Production and Infection

The HCV JFH1 strain was in vitro transcribed using pJFH1 as a template [53] and electroporated into Huh7.5 cells; supernatants were harvested, filtered (0.45 µm), and viral titer quantified using the TCID50 method [54]. Huh7.5 cells were infected at an MOI of 0.1 at day 0. At day 3, when replication generally reached maximal levels, cultures were transfected with si/sh RNAs/vectors to inhibit/knock out Cyp activity and expression. Cells were harvested an additional 5 days later to assess the effect of Cyp knock down/inhibition on viral replication. 

For HBV particle production, HepAD38 culture medium was collected and combined with 4% PEG 8000 (Sigma-Aldrich/Merck St. Louis, MO, USA, incubated at 4 °C overnight on a shaker, and then centrifuged at 4000× *g* for 1 h at 4 °C to pellet the virus. The supernatant was discarded and the pellet resuspended in medium and the titer determined by qPCR (genome copies/mL) as previously described [55]. For infection, on day 1, HepG2-hNTCP or PHHs were seeded in collagen-coated 12-well plates at 3 × 10^5^ cells/well in DMSO or Williams based medium, respectively. On day 2, 2.5% DMSO was added. On day 3, the cells were infected at a MOI of 250 in the presence of 4% PEG8000, incubated for 16 h, and then the PEG-containing inoculum was removed by washing. Cells were maintained in medium containing 2.5% DMSO. On day 7, the cultures were transfected with si/sh RNAs/vectors.

### 2.3. ELISA

HBeAg and HBsAg concentrations were determined using chemiluminescence immunoassay kits from AUTOBIO according to manufacturer’s protocols.

### 2.4. Transfection

Transfections of siRNA (10 pmol/12well) or shRNAs vectors (Table 1 and Table 2) were performed with RNAimax (ThermoFisher Scientific, Waltham, MA, USA) and JetPrime (TebuBio, Le Perray en Yvelines, France), respectively, according to the manufacturer’s protocol.

### 2.5. Western Blot Analysis and Immunofluorescence

Cell cultures were lysed with RIPA Buffer. A total of 30 µg of protein lysate were separated by 15% SDS- polyacrylamide gel electrophoresis, transferred onto nitrocellulose membranes, and blocked with 5% (*w*/*v*) non-fat milk in phosphate-buffered saline with 0.1% Tween-20 for 1 h and incubated with a mouse monoclonal primary antibody against anti-PPIA (cat. no. WH0005478M1; Sigma), rabbit polyclonal anti-CypB (cat. no. PA1-027A Life technologies), rabbit polyclonal anti-PPIC (cat. no. HPA039163; Sigma), rabbit polyclonal anti-PPIC (cat. no. ab184552; Abcam, Cambridge, UK), mouse monoclonal anti-CypD (cat. no. ab110324; Abcam), mouse monoclonal anti-PPIG (cat. no. SAB1404709 and WH0009360; Sigma/Merck St. Louis, MO, USA), or rabbit polyclonal anti-PPIH (cat. no. PA5-31134; Life technologies; cat. No. ab151216 Abcam, Cambridge, UK) overnight at 4 °C. A mouse monoclonal β-actin antibody was used as a loading control (cat. no. A5316; Sigma/Merck St. Louis, MO, USA). Following incubation with the corresponding horseradish peroxidase-conjugated anti-mouse secondary antibodies (cat. no. A4416; Sigma/Merck St. Louis, MO, USA) or anti-rabbit secondary antibody (cat. no. A6154; Sigma/Merck St. Louis, MO, USA) at room temperature for 1 h, the protein bands were visualized by using enhanced chemiluminescence (Clarity Western ECL; Bio-Rad, Hercules, CA, USA). The optical density of the protein bands was quantified using ImagLab software (Bio-Rad, Hercules, CA, USA).

Immunofluorescence was performed as previously described [56] using an HCV-patient serum or anti-HCV core (SCBT C-750, SCBT, Dallas, TX, USA) at 1:200.

### 2.6. RT/qPCR for HCV and HBV Quantification

Total intracellular RNA was extracted using Extract-All (Eurobio, Les Ulis, France) according to the manufacturer’s protocol. A total of 1 µg of total RNA was DNase-treated (Roche, Basel, Switzerland) and reverse transcribed (M-MLV RT, ThermoFisher Scientific, Waltham, MA, USA). HCV RNA quantification was performed by quantitative reverse transcription-PCR (RT-qPCR) using SYBR Green I Master Mix (Roche, Basel, Switzerland) on a LightCycler 480 (Roche, Basel, Switzerland). Glucuronidase beta (GUS) served as a reference gene (5′-CGTGGTTGGAGAGCTCATTTGGAA-3′, 5′-ATTCCCCAGCACTCTCGTCGGT-3′). The HCV primer sequences used were 5′-gtctagccatggcgttagta-3′ and 5′ ctcccggggcactcgcaagc-3′. Total intracellular DNA was extracted using MasterPure DNA extract (Euromedex, Souffelweyersheim, France) according to the manufacturer’s protocol. HBV DNA quantification was performed by qPCR using SYBR Green I Master Mix (Roche) on a LightCycler 480 (Roche) with 20 ng of total DNA. RPLPO served as a reference gene (5′-CACCATTGAAATCCTGAGTGATGT-3′, 5′-TGACCAGCCCAAAGGAGAAG-3′). For HBV, the primer sequences used were 5′-GCTGACGCAACCCCCACT-3′ and 5′ AGGAGTTCCGCAGTATGG-3′.

### 2.7. Statistics

Data are expressed as mean ± standard deviation (SD). Statistical significance was determined by analysis of variance (ANOVA) with Tukey’s post hoc test or Dunnett’s test using Graphpad Prism (10.0). A value of *p* < 0.05 was considered statically significant (* *p* < 0.05, ** *p* < 0.01, and *** *p* < 0.001). 

## 3. Results

### 3.1. Cyclophilin G and H Are Required for Efficient HCV Replication

**Figure 1:**
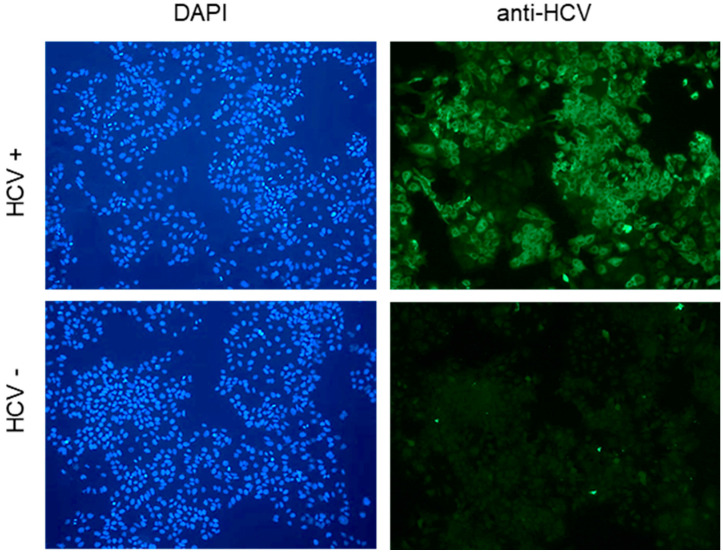
HCV infection at 3 days post infection. Huh7.5 cells were infected with HCV at an MOI of 0.1 and fixed for immunofluorescence analysis 3 days later. Green: anti-HCV patient serum; blue: DAPI staining. A representative image is shown; scale bar: 50 µm.

To ask what Cyps may impact replication in vitro in a setting of an already-established productive HCV infection, we screened siRNAs and shRNAs targeting CypA, B, C, D, G, and H. Huh7.5 cells were infected with the virus at an MOI of 0.1 and cultured for 3 days. At day 3, spread of HCV throughout the culture was validated by immunofluorescence (Figure 1), and the cultures were transfected with either of two different siRNAs targeting a given Cyp or a scramble control, and viral replication was assessed 5 days later by RTqPCR. 

**Figure 2:**
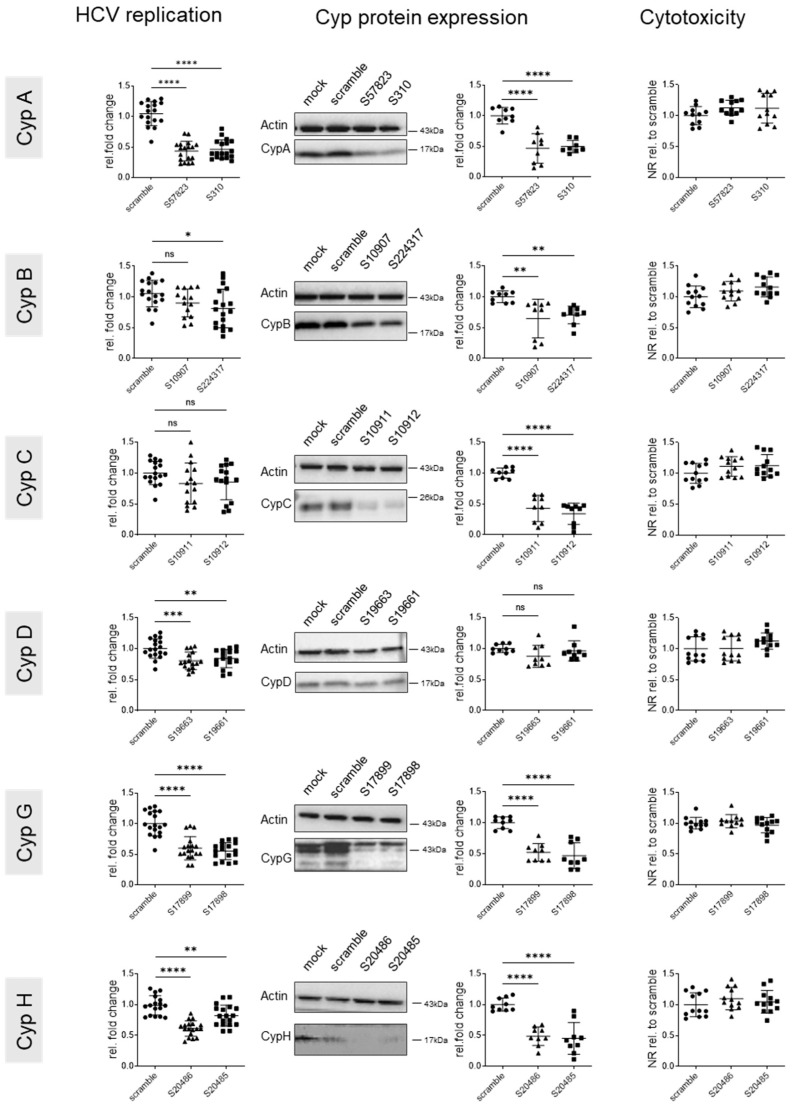
Effect of genetic cyclophilin knock down (siRNA) on intracellular HCV replication. Huh7.5 cells were infected at an MOI of 0.1. Then, 72 h later, when HCV replication was well established, cells were transfected with siRNAs targeting the indicated Cyps and harvested 5 days after transfection. Left panels: intracellular HCV replication determined by RTqPCR with GUS as housekeeping gene and standardized to scramble controls. Middle panels: Cyp expression was assessed by Western blotting, quantified using ImageJ, standardized to actin, and data were expressed relative to the scramble conditions. Representative Western blots are shown. Right panels: cytotoxicity was assessed by neutral red staining with data standardized to the scramble control. Means +/− STD, *n* = 3, with each experiment performed with at least three culture wells and each well representing a datapoint. One-way anova. * *p* < 0.05, ** *p* < 0.01, and *** *p* < 0.001; **** *p* < 0.0001.

In the absence of detectable cell toxicity at the moment of harvest, HCV replication was sensitive to knock down of CypA (Figure 2). No effect of CypB and C and only a very marginal effect of CypD knock down on viral replication could be observed—in the cases of CypB and D this is very likely due to the low knock down efficacy of the siRNAs. Knock down of both CypG and H reduced HCV replication in a reproducible manner; although, the effect was overall less important than that observed upon CypA knock down. Importantly, the knock down of CypA, D, G, and H did not affect expression levels of other Cyps, suggesting that the observed effects were specifically mediated by the targeted Cyp (Supplementary Figure S1).

**Figure 3:**
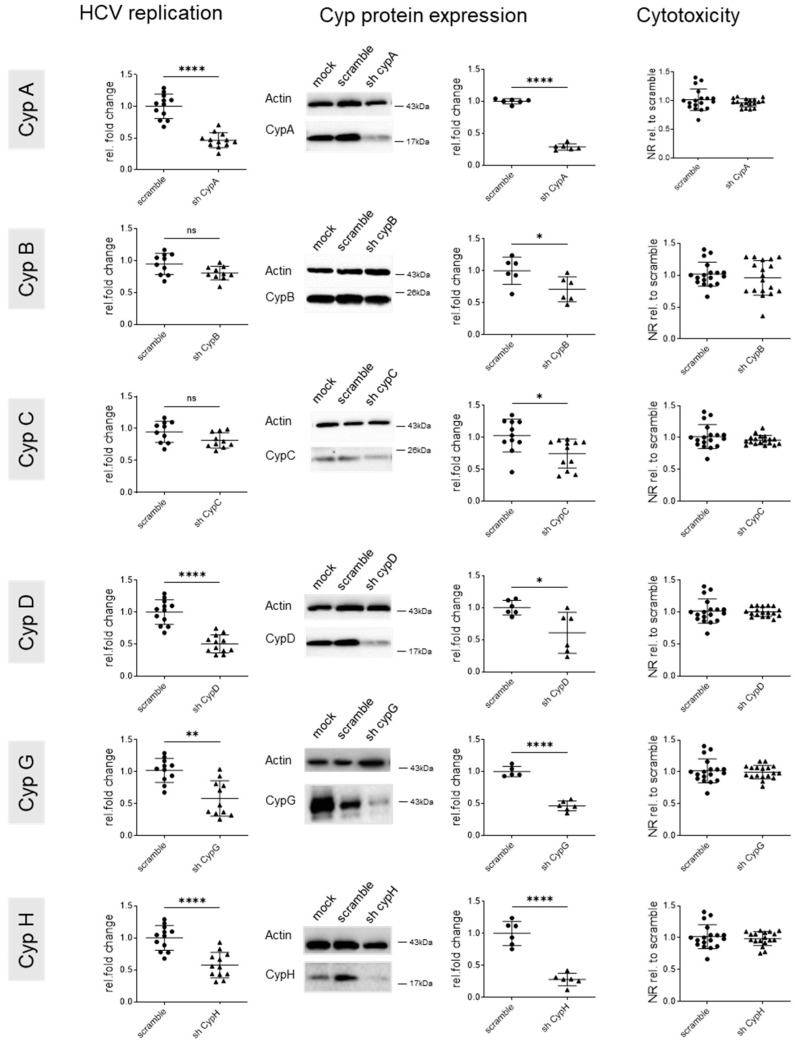
Effect of genetic cyclophilin knock down (shRNA) on intracellular HCV replication. Huh7.5 cells were infected at an MOI of 0.1. Then, 72 h later, when HCV replication was well established, cells were transfected with shRNA vectors targeting the indicated Cyps and harvested 5 days after transfection. Left panels: intracellular HCV replication was determined by RTqPCR using GUS as a housekeeping gene. Data were standardized to HCV RNA levels observed in scramble controls. Middel panels: Cyp expression was assessed by Western blotting, quantified using ImageJ, standardized to actin, and data were expressed relative to the scramble conditions. Representative Western blots are shown. Right panels: cytotoxicity was assessed by neutral red staining with data standardized to scramble control. Means +/− STD, *n* = 3, with each experiment performed with at least two culture wells and each well representing a datapoint. One-way anova. * *p* < 0.05, ** *p* < 0.01, and **** *p* < 0.0001.

These data were confirmed by transfecting shRNA encoding vectors encoding target sequences different to those of the siRNAs, using the same experimental layout (Figure 3). Again, a strong inhibition of HCV replication was observed with an shRNA targeting CypA, and no significant effects were observed with shRNAs targeting CypB or C. However, for CypB, knock down efficacy was again very low. Knock down of CypD, G, and H did impact HCV replication. This suggests that targeting cyclophilins A, D, G, and H blocks not only HCV neo-infections, as already reported, but also established HCV infection.

### 3.2. Knock Down of CypA Impacts Established HBV Infection

**Figure 4:**
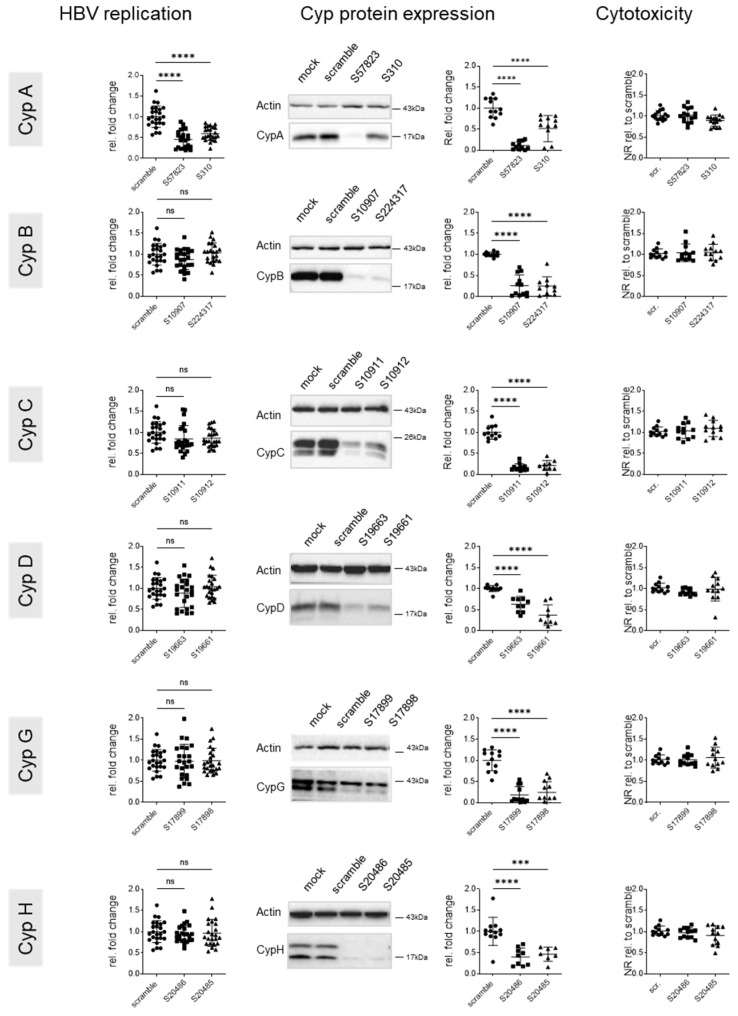
Effect of genetic cyclophilin knock down on intracellular HBV replication. HepG2-hNTCP cells were seeded at 4.10^5^/well into 12 wells; cells were infected at day 3 at an MOI of 100 and transfected at day 10 with the indicated siRNAs. Cells were harvested 5 days after transfection to assess HBV replication and Cyp expression. Left panel: intracellular HBV replication was determined by qPCR using RPLP0 as a housekeeping gene. Results are expressed relative to scramble; middle panel: Cyp expression was assessed by Western blotting, quantified using ImageJ, standardized to actin, and data were expressed relative to the scramble conditions. Representative Western blots are shown. Right panel: cytotoxicity was assessed by neutral red staining with data standardized to the scramble control. Means +/− STD, *n* = 3, with each experiment performed with a minimum of three culture wells and each well representing a datapoint. One-way anova. *** *p* < 0.001 and **** *p* < 0.0001.

**Figure 5:**
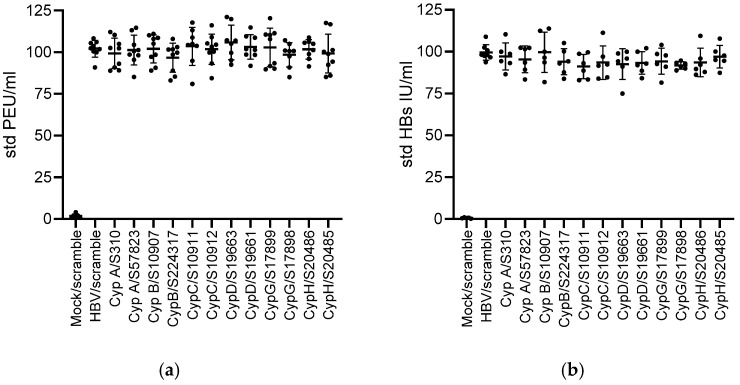
HBs and HBe antigen secretion are not affected by Cyp knock down. HepG2-hNTCP cells were seeded at 4.10^5^/well into 12 wells, DMSO was added 1 day later for 24 h. Cells were then infected at an MOI of 100 and transfected 7 days later with the indicated siRNAs. Cell supernatants were harvested at the time of transfection at day 7 and 5 days after transfection to assess (**a**) HBe (PEIU/mL) and (**b**) HBs (UI/mL) antigen secretion. Data were standardized to values obtained for the HBV/scramble control at day 7 (time of transfection). Means +/− STD, *n* = 5. One-way anova: no statistically significant difference was observed between the conditions.

HBV entry as well intracellular replication in the context of neo-infections have previously been shown to be sensitive to the knock down of several Cyps. In order to assess the role of particular Cyps in established HBV replication, HepG2-hNTCP cultures were infected with HBV at an MOI of 100 and cultured for 7 days. Seven days after infection, productive replication was verified by quantification of HBs and HBe antigens in supernatants. Cultures were transfected with siRNAs targeting cyclophilin expression the same day, i.e., day 7 post infection. The impact of Cyp knock down on viral replication was assessed by harvesting cultures 5 days after transfection and quantifying HBs and HBe antigens in supernatants and total intracellular DNA in cell lysates (Figure 4 and Figure 5). 

siRNA treatment did not show any cytotoxic effects on the cultures. Only knock down of CypA had a significant effect on intracellular HBV DNA levels, with the siRNA resulting in the higher CypA knock down efficacy (S57832) and also showing are more pronounced reduction in HBV DNA. Knock down of CypA was specific, as expression of none of the other tested Cyps was affected (Supplementary Figure S2). No effect on HBV was observed with siRNAs targeting any other Cyp, despite good knock down efficacies (Figure 4). No effects on HBs and HBe antigen secretion were noted (Figure 5).

**Figure 6:**
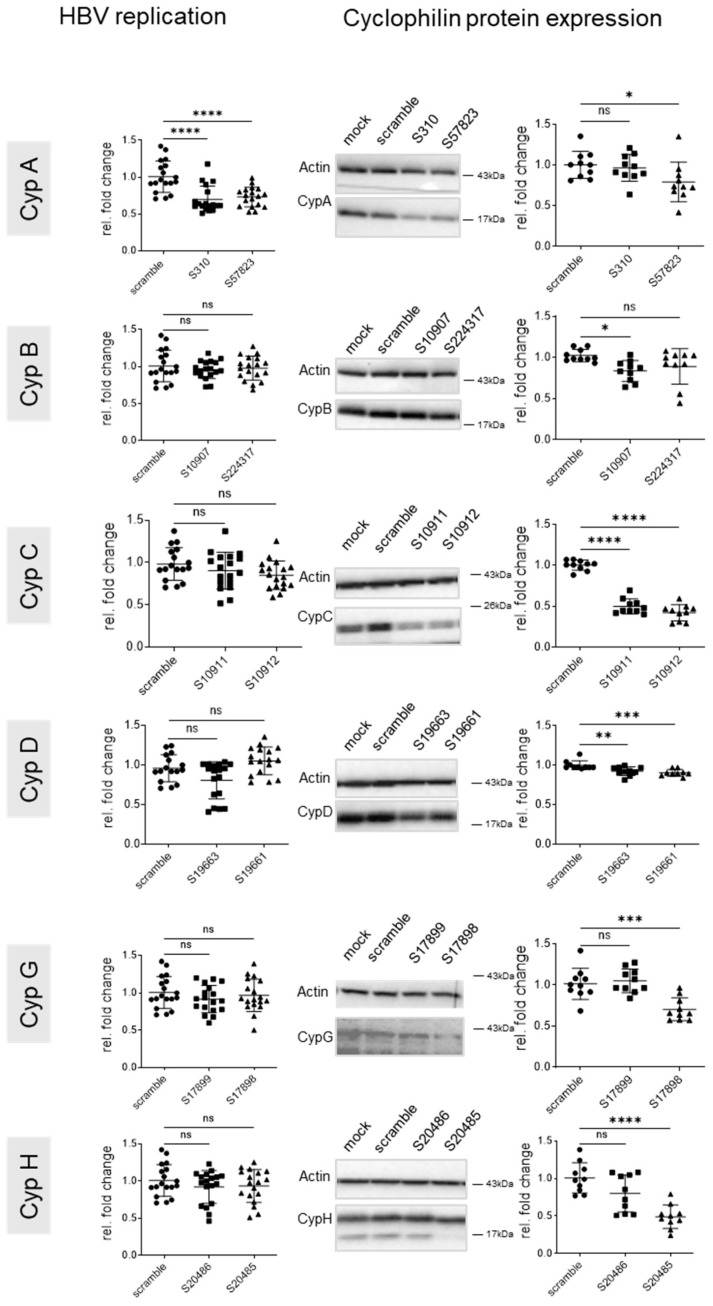
Effect of genetic Cyp knock down on intracellular HBV replication in PHH. PHHs were infected (MOI 250) and transfected 7 days later with the siRNAs targeting the indicated Cyps. Cells were harvested 5 days after transfection. Left panels: intracellular HBV replication quantified by qPCR with RPLP0 as a housekeeping gene. Right panels: Cyclophilin expression by Western blotting, quantified with ImageJ, standardized to actin, and data were expressed relative to the scramble. Representative Western blots are shown. Means +/− STD, *n* = 3, with each experiment performed with at least three culture wells with each well representing a datapoint. One-way anova. * *p* < 0.05, ** *p* < 0.01, *** *p* < 0.001 and **** *p* < 0.0001.

**Figure 7:**
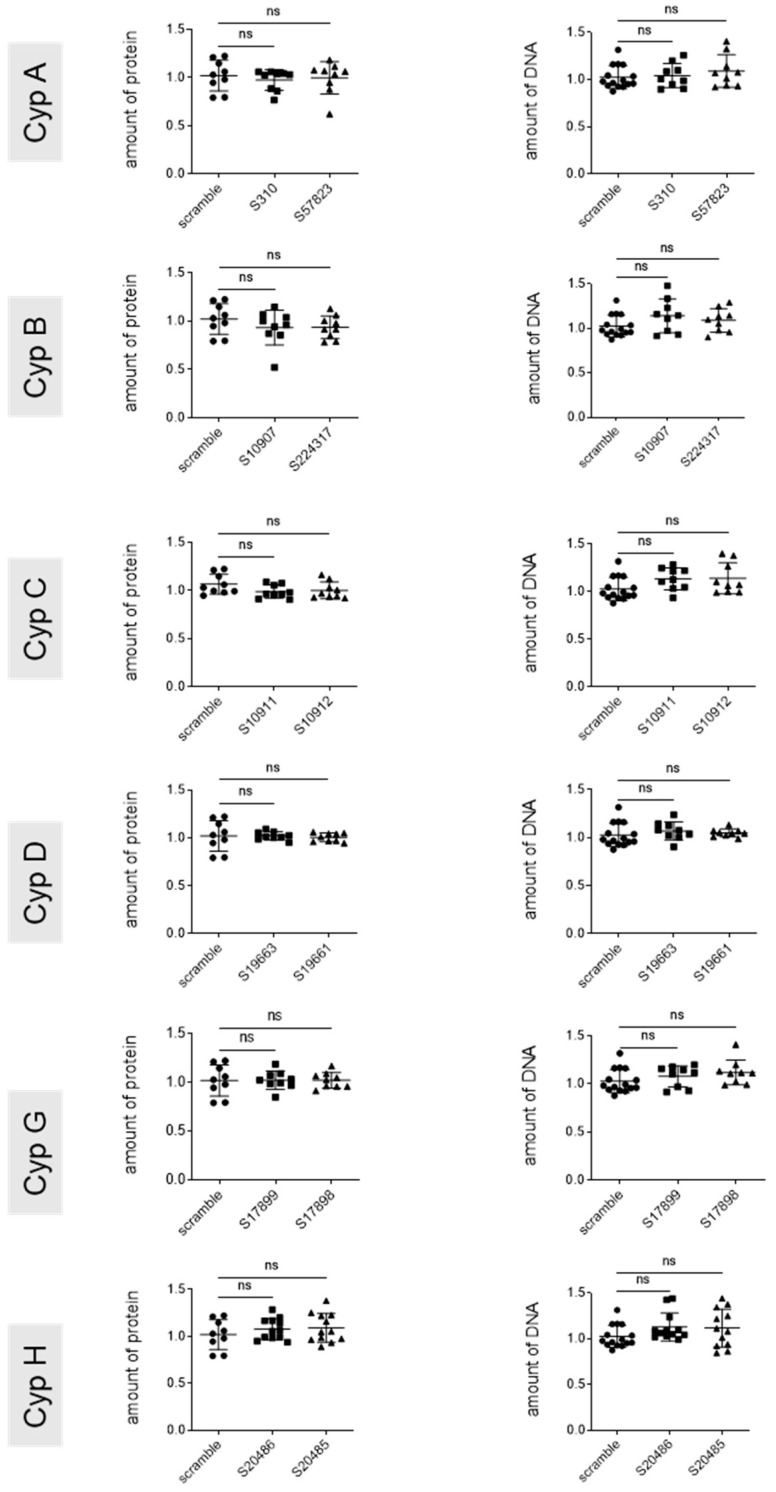
Cytotoxicity of genetic cyclophilin knock down by siRNA. PHHs were seeded at 1.10^6^/well into 12 wells. DMSO was added 1 day later for 24 h. Cells were then infected at an MOI of 250 and transfected 7 days later with the indicated siRNAs. Cells were harvested 5 days after transfection. Total protein (**left panel**) and DNA (**right panel**) were quantified as the read out for cell toxicity. *n* = 3, means +/− STD, with each experiment performed with at least three culture wells with each well representing a datapoint; One-way anova.

**Figure 8:**
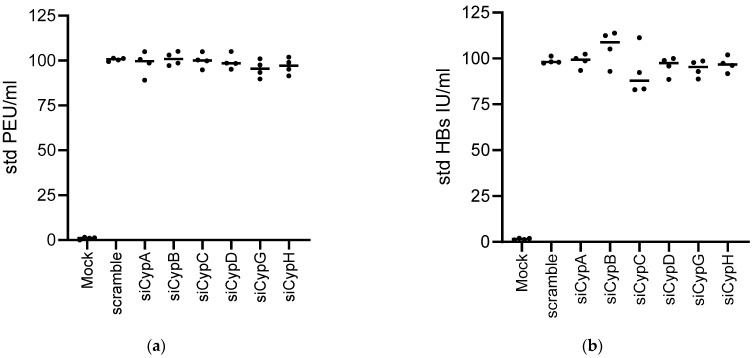
HBs and HBe antigen secretion are not affected by Cyp knock out. PHHs were seeded at 1.10^6^/well into 12 wells. DMSO was added 1 day later for 24 h. Cells were then infected at an MOI of 250 and transfected 7 days later with the mixtures of siRNAs (as used in Figure 6), targeting the indicated siRNAs. Cell supernatants were harvested at the time of transfection at day 7 and 5 days after transfection to assess (**a**) HBe (PEIU/mL) and (**b**) HBs (UI/mL) antigen secretion. Data were standardized to values obtained for the HBV/scramble control at day 7 (time of transfection). Means +/− STD, *n* = 4. One-way anova: no statistically significant difference was observed between the conditions.

Assessment of the effect of siRNAs on primary human hepatocytes (PHHs) infected with HBV using the same experimental layout (7-day infection followed by transfection and harvest 5 days after transfection) showed similar results (Figure 6 and Figure 7). The effect of CypA knock down on HBV replication was attenuated in comparison to HepG2-hNTCP cells but reproducible, most likely because the knock down efficacy of CypA in PHH was less efficient compared to HepG2-hNTCP. Unfortunately, it was not possible to assess cell viability by neutral red staining due to the limited amount of PHH available for the experiment, but no effect of the siRNAs on total protein or DNA yields was noted in these cultures, suggesting the absence of cytotoxic effects (Figure 7). Again, no effect on HBs and HBe antigen secretion was noted (Figure 8). The levels of these antigens remained similar to those detected before siRNA transfection, which also argues for the lack of cell toxicity of the siRNAs.

## 4. Discussion

The physiological functions of Cyps are implicated in various different pathologies including liver diseases. A number of non-immunosuppressive pan-CypIs have been described in the past with anti-viral or other therapeutic activities. However, the cyclophilin family members that play the most important role in the progression of disease and therapeutic intervention have often remained elusive. Efforts are currently undergoing towards the development of specific CypIs, targeting for example CypD for the treatment of neurodegenerative diseases [57]. In parallel, use of transgenic approaches has shown that CypB is a crucial target in the treatment of liver disease with the pan-cyclophilin inhibitors CRV431, a CsA analog, and NV556, a derivative of sangliferhin A [58]. 

In the context of viral hepatitis, pan-CypIs have proven to be of therapeutic value to treat chronic infections for hepatitis C [24,25,26], while in a transgenic mouse line carrying the HBV genome, the pan-cyclophilin inhibitor CRV431 reduced liver HBV DNA levels and moderately reduced serum HBsAg levels [31]. Aiming to decipher the underlying molecular mechanisms and in particular the specific Cyps involved, several laboratories have identified CypA as key factor involved in polyprotein processing, replication, and double-membrane vesicle formation in HCV infection [8,10,32,33,34,35,36,37,38,39,40]. Over time, additional cyclophilins have been implicated in the HCV life cycle, even though the molecular details remain unknown [8,9,10,41,42,43,44,45,46,47]. However, many of these reports have addressed the roles of particular Cyps in neo-infections, where the genetic knock down was performed before or concomitantly with HCV inoculation. Few studies have targeted expression of specific Cyps in the context of an established/chronic productive infection and assessed the impact on viral replication. In the context of HBV infection, the studies are less advanced. A therapeutic effect in vivo has been shown in the context of a transgenic mouse model stably expressing an HBV genome [31]. In vitro studies have implied roles for CypA, CypC, and CypD [29] but, again, not in a context of an established, in vitro infection system. 

We have used si- as well as sh- approaches targeting Cyps A, B, C, D, G, and H, with results of both approaches being overall consistent. We confirm that inhibition of CypA and D, in the context of an established infection, has antiviral effects on HCV replication. This underlines the importance of CypA in double-vesicle formation, replication, and proteolytic processing of the viral protein precursor. In contrast, knock down of CypB, also known to interact with HCV viral proteins, did not result in a reduction in viral replication. However, downregulation of CypB in Huh7.5 cells with si- as well as shRNA approaches was very inefficient. Better knock down efficacies will be necessary to further assess the antiviral role of CypB. To this end, additional siRNAs (smartpool Dharmakon) targeting CypB in Huh7.5 cells were tested, but without much success. Knock down of CypD, important for the formation of mitochondria-associated membranes (MAMs), to which several HCV proteins localize [46], also impacted HCV replication considerably. However, the exact role of CypD and MAM in the viral life cycle remains to be elucidated. Furthermore, we have identified CypG and CypH as additional cyclophilins that play a role in HCV replication. CypH, but not CypG or CypD knock down, have previously been shown to affect replication levels of subgenomic HCV replicons [45]. Whether this discrepancy is due to differences in culture system or kinetics remains unclear. Both CypG and CypH display nuclear localization and associate with the spliceosome. Using a spliceosome-wide approach based on siRNA pools, it has been reported that especially PPIH actively participates in regulating the splicing of targets in apoptosis and inflammation [5]. Besides CypG and CypH, additional human Cyps (e.g., CypE, PPWD1, PPPIL1, and PPIL3) are known to bind to spliceosomal complexes as well as associated factors at different stages of assembly, suggesting that nuclear Cyps are distributed throughout the splicing cycle in order to play some regulatory roles [59]. Interestingly, HCV replication is known to depend on the splicing factor serine-arginine-rich protein kinases (SRPKs) [60] and is regulated by interferon response genes whose expression is regulated by the spliceosome factor SART1 [61]. However further studies are needed to shed light on the potential roles of CypG and CypH in splicing and the impact on HCV replication.

The role of cyclophilins in the life cycle of HBV has been predominantly assessed in the context of neo-infections or immediately after infection [13,29]. In this context, CypIs have been shown to inhibit HBV replication and HBs production [29,31] and HBV entry [13]. Phillips et al. showed, furthermore, that alisporivir reduced intracellular and secreted HBV DNA, while knock down of CypA, CypC, and CypD all reduced HBV DNA and secreted HBsAg levels in HepG2 cells stably expressing an HBV genome [29]. Finally, a pan-cyclophilin inhibitor reduced intrahepatic HBV DNA in HBV-transgenic mice [13]. However, in vivo studies that investigate the therapeutic effect of CypIs in the context of chronic hepatitis B are missing. As a first step in this direction, we have investigated the effects of Cyp silencing in vitro in a therapeutic setting where treatments were applied to PHH or HepG2^NTCP^ 7 days post infection, once HBV replication was well established. Genetic knock down of individual Cyps had only a moderate or no effect on total HBV DNA or HBs and HBe antigen secretion, respectively. Only knock down of CypA moderately decreased HBV DNA replication in both HepG2NTCP cells as well as PHH. Even extension of the analysis to 8 days post transfection, to render the knock down effect of the siRNAs potentially more efficient, did not impact HBs and HBe antigen secretion. In contrast to Phillips et al. [29], we did not observe an effect of CypA knock down on HBs or HBe antigen secretion, nor did we observe antiviral effects of CypC and CypD knock down. Whether this is due to differences in the knock down efficacies of the siRNAs targeting CypA, C, or D or other reasons remains to be elucidated. The moderate effect of CypA silencing, which we observed, is consistent with reports that show that CypA knock down reduces recycling of HBV nucleocapsids from the cytoplasm to the nucleus, a key mechanism for maintaining or replenishing the pool of intranuclear covalently closed circular HBV DNA [29]. 

Future studies, where Cyp expression and functions are inhibited in already-infected cultures or animal models, or concomitantly with the virus, are much needed and will enable the validation of whether the so-far reported anti-HBV activity of CypIs is mediated solely by CypA.

## Figures and Tables

**Table 1:** Commercial siRNA constructs used in this study.

Nomenclature	Target	Reference
silencer siRNA PPIA ID:s310	Hs peptidylprolylisomerase A (PPIA)	4392420
silencer siRNA PPIA ID:s57823	Hs peptidylprolylisomerase A (PPIA)	4392420
silencer select validated siRNA PPIB ID:s10907	Hs peptidylprolylisomerase B (PPIB)	4390824
silencer select validated siRNA PPIB ID:s224317	Hs peptidylprolylisomerase B (PPIB)	4390824
silencer select validated siRNA PPIC ID:s10911	Hs peptidylprolylisomerase C (PPIC)	4390824
silencer select validated siRNA PPIC ID:s10912	Hs peptidylprolylisomerase C (PPIC)	4390824
silencer select validated siRNA PPIF ID:s19661	Hs peptidylprolylisomerase F (PPIF) *	4390824
silencer select validated siRNA PPIF ID:s19663	Hs peptidylprolylisomerase F (PPIF) *	4390824
silencer siRNA PPIG ID:s17898	Hs peptidylprolylisomerase G (PPIG)	4392420
silencer siRNA PPIG ID:s17899	Hs peptidylprolylisomerase G (PPIG)	4392420
silencer select validated siRNA PPIH ID:s20485	Hs peptidylprolylisomerase H (PPIH)	4390824
silencer select validated siRNA PPIH ID:s20486	Hs peptidylprolylisomerase H (PPIH)	4390824

* The gene named PPIF encodes CypD.

**Table 2:** Commercial shRNA constructs used in this study.

Nomenclature	Target	Reference
NM_021130/TRCN0000049232/PLKO.1	Hs peptidylprolylisomerase A (PPIA)	TRCN0000049232
NM_000942/TRCN0000296764/PLKO.1	Hs peptidylprolylisomerase B (PPIB)	TRCN0000296764
NM_000943/TRCN0000049253/PLKO.1	Hs peptidylprolylisomerase C (PPIC)	TRCN0000049253
NM_005729/TRCN0000232682/PLKO.1	Hs peptidylprolylisomerase F (PPIF)	TRCN0000232682
NM_006347/TRCN0000289678/PLKO.1	Hs peptidylprolylisomerase G (PPIG)	TRCN0000289678
NM_004792/TRCN0000049326/PLKO.1	Hs peptidylprolylisomerase H (PPIH)	TRCN0000049326

## Data Availability

The original images and western blots presented in this study are included in the supplementary material. Request for raw data concerning PCR or ELISA based assays can be directed to the corresponding author(s).

## Supplementary Materials

The following supporting information can be downloaded at https://www.mdpi.com/article/10.3390/v17010011/s1.
