# Supplementary material for: Exploration of the Role of Cyclophilins in Established Hepatitis B and C Infections
Source: Viruses. 2024 Dec 25;17(1):11. doi: 10.3390/v17010011 (PMC11768883; doi:10.3390/v17010011)
Supplement: Supplementary file 1 [file viruses-17-00011-s001.zip › viruses-3071202-supplementary.pdf]

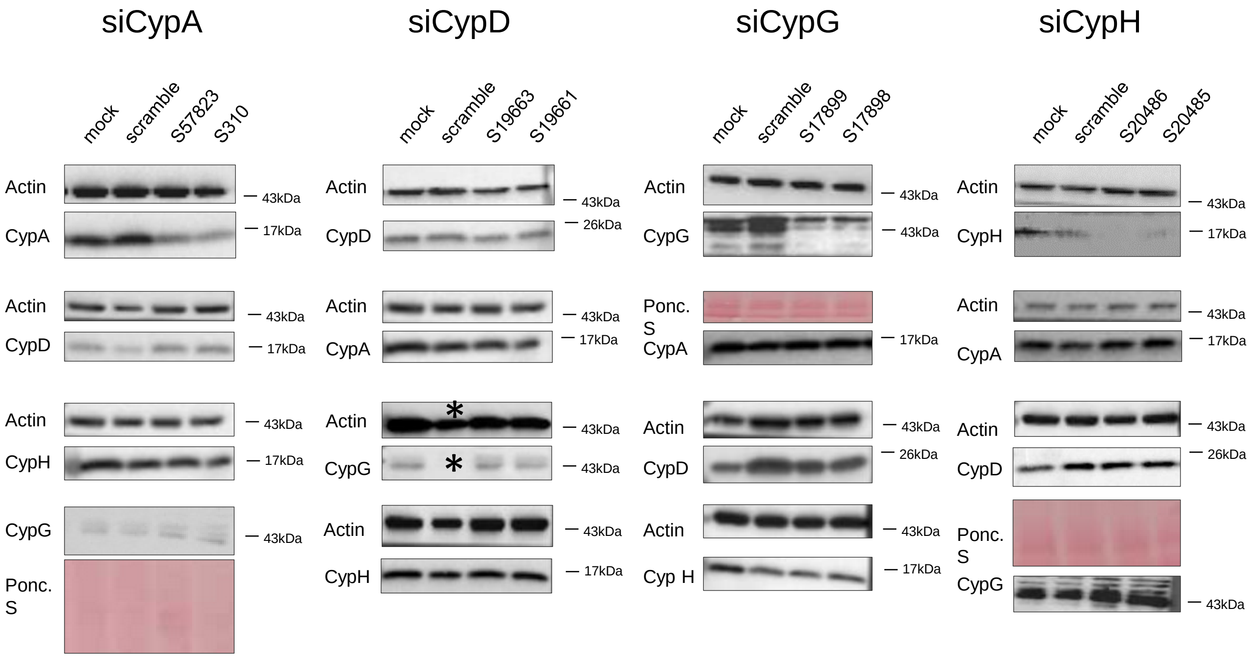

Supplementary Figure S1. Cell lysates used for the westernblots for conditions siCypA, siCypD, siCypG and siCypH depicted in Figure 2 were reblotted with antibodies targeting Cyclophilins A, D, G and H as indicated. Where control blots with anti-actin were not possible to perform, a ponceau S staining is shown as loading control. * less cell lyaste was loaded due to lack of remaining sample.

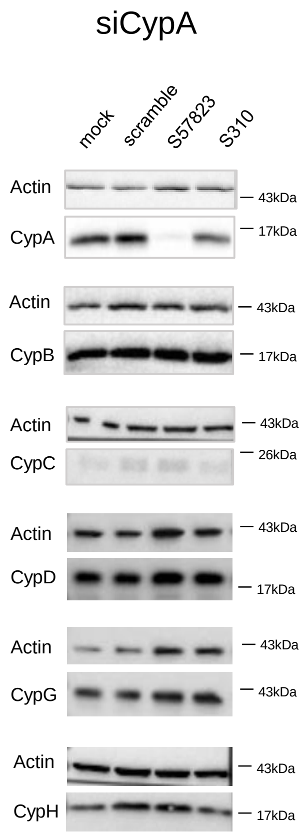

Supplementary Figure S2. Cell lysates used for the westernblots for the condition siCypA depicted in Figure 4 were reblotted with antibodies targeting Cyclophilins A, D, G and H.
